# Potential Utilization of Ground Eggshells as a Biofiller for Natural Rubber Biocomposites

**DOI:** 10.3390/ma16082988

**Published:** 2023-04-09

**Authors:** Anna Sowińska-Baranowska, Magdalena Maciejewska

**Affiliations:** Department of Chemistry, Institute of Polymer and Dye Technology, Lodz University of Technology, Stefanowskiego Street 16, 90-537 Lodz, Poland

**Keywords:** biocomposites, natural rubber, biofillers, eggshells, silanes, ionic liquids, ammonium surfactant

## Abstract

The aim of this work was application of ground eggshells in various amounts by weight as a biofiller for natural rubber (NR) biocomposites. Cetyltrimethylammonium bromide (CTAB), ionic liquids (ILs), i.e., 1-butyl-3-methylimidazolium chloride (BmiCl) and 1-decyl-3-methylimidazolium bromide (DmiBr), and silanes, i.e., (3-aminopropyl)-triethoxysilane (APTES) and bis [3-(triethoxysilyl)propyl] tetrasulfide (TESPTS), were used to increase the activity of ground eggshells in the elastomer matrix and to ameliorate the cure characteristics and properties of NR biocomposites. The influence of ground eggshells, CTAB, ILs, and silanes on the crosslink density, mechanical properties, and thermal stability of NR vulcanizates and their resistance to prolonged thermo-oxidation were explored. The amount of eggshells affected the curing characteristics and crosslink density of the rubber composites and therefore their tensile properties. Vulcanizates filled with eggshells demonstrated higher crosslink density than the unfilled sample by approximately 30%, whereas CTAB and ILs increased the crosslink density by 40–60% compared to the benchmark. Owing to the enhanced crosslink density and uniform dispersion of ground eggshells, vulcanizates containing CTAB and ILs exhibited tensile strength improved by approximately 20% compared to those without these additives. Moreover, the hardness of these vulcanizates was increased by 35–42%. Application of both the biofiller and the tested additives did not significantly affect the thermal stability of cured NR compared to the unfilled benchmark. Most importantly, the eggshell-filled vulcanizates showed improved resistance to thermo-oxidative aging compared to the unfilled NR.

## 1. Introduction

Various additives are used in elastomeric composites to achieve the properties required for specific rubber product applications. Among those additives, fillers are one of the most crucial and the second largest following the rubber and the crosslinking system [1]. The most common fillers of elastomer composites are chalk; silica; carbon fillers, including carbon black, graphite, and carbon fibers; as well as silicates, i.e., talc, kaolin, and others [2]. The addition of reinforcing filler usually increases the modulus and results in the significant improvement in the mechanical properties of elastomer composites, including their abrasion and tear resistance. On the other hand, non-reinforcing fillers could cause deterioration of strength properties but may increase hardness and modulus of the composite. Moreover, they are usually used as extenders or diluents in order to lower the cost of manufacturing rubber products [3].

Nowadays, one of the most popular and widely used fillers of inorganic origin is calcium carbonate (CaCO_3_). It is widely applied in thermoplastics, thermosets, and elastomers. CaCO_3_ used as a filler can be of both natural and synthetic origin (precipitated CaCO_3_). In nature, CaCO_3_ protrudes in large deposits near the earth’s surface. Therefore, it is easy to mine it. In order for the natural CaCO_3_ to be used as a filler, it must be ground, dried, and purified. It is also recommended to treat this filler, e.g., with fatty acids, in order to hydrophobize the surface of the finely milled particles and consequently make them well dispersed in the elastomer matrix [4].

Another natural and easily available source of CaCO_3_ can be chicken eggshells, which every year are produced in plenty as a waste material. In developed countries, nearly a third of the eggs produced are sent to breakers to produce liquid eggs. This results in a large amount of biowaste in the form of eggshells [5]. The disposal of such a large amount of waste has become a challenging problem, and therefore scientists give prime importance for their management. Due to the eggshells being cheap and lightweight, they could be used in load-bearing materials, e.g., for the automotive industry and various constructive uses [6].

Ummartyotin et al. [7] have examined the utilization of CaCO_3_ from waste eggshells as a reinforcement for bacterial cellulose composite membranes. Applying milled eggshells improved flexibility and thermal stability of the composites but deteriorated their tensile strength. Some biotechnological applications of waste eggshells are also widely known, including patented inventions for biomedical, engineering, chemical, and environmental technologies [8,9,10,11].

Generally, the white side of the eggshells consists of membranes with tightly interwoven fibers. The internal of these two membranes remains uncalcified, whereas the external one undergoes a partial mineralization [12,13,14]. Porous eggshell enables exchanging of water and metabolic gases. Regarding chemical nature, eggshells consist mainly of calcium carbonate crystals (~90%), which are referred to as calcite, and organic matter from membranes. The remaining components represent minerals, including magnesium (Mg), phosphorus (P), potassium (K), sodium (Na), silicon (Si), zinc (Zn) in trace amounts, and some carbon groups. The presence of alkali metals, i.e., K, Na, Ca, in eggshells seems to be important since they have been reported to improve the scorch safety during processing of rubber compounds [15,16,17]. Owing to the presence of calcium and organic semi-permeable membranes and pores, eggshells were proposed to be applied as adsorbents or catalysts [5]. It has been reported that calcium-rich eggshells could be also potentially employed as precursors in the selective oxidation of methane, which supplies about 80–90% of the natural gas that is commonly available [18]. Therefore, it could be suspected that calcite as the most crucial component of eggshells, which fills their pores, may be of great importance in the adsorption of ingredients or in the potential interaction with the compounds used in our studies.

The interactions between polymer and filler have a meaningful influence on the properties of vulcanizates. Therefore, to enhance the properties of final rubber products, many filler modifications of a chemical and physical nature are used or modifiers are directly added to the polymer matrix [19,20,21,22,23]. It is generally known that the homogenous dispersion and distribution of particles introduced to the elastomer matrix affects the elastomer-filler interaction and thus the useful parameters of vulcanizates. Several authors have used silanes for enhancing the interfacial interactions and thus the compatibility between elastomers and fillers [24,25,26]. The application of ionic liquids is also known to positively affect the dispersion of filler and curatives and thus the properties of vulcanizates [27,28,29]. Moreover, the crucial importance of alkylammonium salts, i.e., cetyltrimethylammonium bromide (CTAB), has been reported, which acted as a substance enhancing the dispersion of solid additives in polymer matrix and consequently the properties of final products [30,31,32]. However, in many cases these reports from the literature mainly focus on the application of common fillers, i.e., silica, carbon black, or carbon nanofibers.

In recent years, interest in pro-ecological raw materials and biocomposites has been constantly growing, and the use of such materials as an alternative to conventional polymeric materials in various applications plays a fundamental role. Replacing petroleum products with biomass brings many benefits, such as sustainable waste management, recycling of materials, and reducing the supply and demand for products obtained from non-renewable raw materials. The use of biomass as one of the crucial components of elastomeric compounds, i.e., fillers, is widely known [19,20,33,34,35]. One of the current popular solutions from the known state of the art is the production of biocomposites based on the application and management of waste, i.e., nut- or eggshells, vegetable waste, and others, in order to improve, for example, mechanical properties. Miedzianowska et al. [36] have reported elastomeric composites of natural rubber containing rye, oat, or triticale straw with various degrees of grinding and quantitative content as a filler. The authors focused on the management of overproduction of straw and used it as a filler of NR elastomer, which allowed them to obtain product that met the criteria of a biocomposite. The obtained rubber products were characterized by good mechanical properties, high hardness and damping coefficient, as well as good resistance to tearing. Next, suitable example of the using of biomass and waste management is described by Khaigunha et al. [37]. The authors investigated the influence of micron-sized eggshells filler on the resistance to tracking and erosion of silicone rubber composite. Experimental results indicated that the vulcanizates revealed an improvement of tracking and erosion resistance due to an addition of eggshell particles. Furthermore, the amount of eggshells influenced the thermal stability of the composites and proved that higher thermal stability of eggshells affected the heat resistance of the elastomer matrix.

The novelty of this work concerns the utilization of ground waste eggshells in elastomer composites to find a good solution for the waste management and also to improve the strength and properties of the prepared composites, e.g., poor resistance of the NR to thermo-oxidative aging. Moreover, in our studies the validity of the use of various compounds, such as ionic liquids, surfactants, and silanes was studied to improve the vulcanization parameters and additionally to affect the functional properties of the final rubber composites. The whole point of the work is in line with the global trend of waste management to produce biocomposites, which are not only environmentally friendly but also have good functional properties. As mentioned, the disposal of large amount of biowaste in the form of eggshells has become a challenging problem, and therefore it is highly reasonable to find new opportunities for their industrial use. Due to their similar composition to CaCO_3_ and the fact that ground eggshells do not require special treatment to be used in rubber compounds, using them as a filler may be an excellent way to manage this bio-waste.

## 2. Materials and Methods

### 2.1. Materials

**Figure 1:**
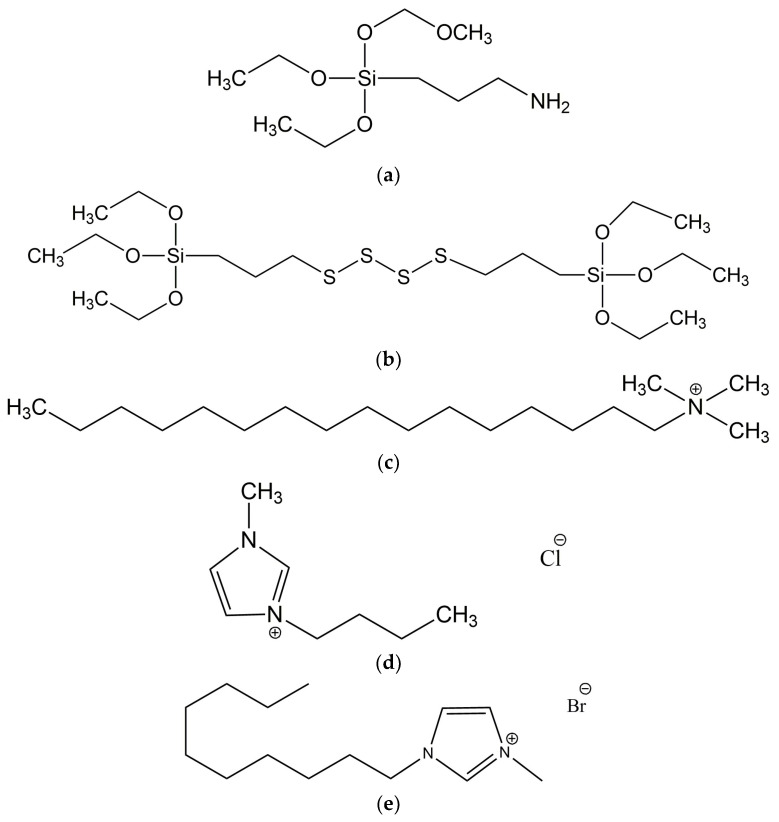
Structure of the additives employed to improve the dispersion degree of ground eggshells in the elastomer matrix: (**a**) (3-aminopropyl)-triethoxysilane, APTES; (**b**) bis[3-(triethoxysilyl)propyl] tetrasulfide, TESPTS; (**c**) cetyltrimethylammonium bromide, CTAB; (**d**) 1-butyl-3-methylimidazolium chloride, BmiCl; (**e**) 1-decyl-3-butylimidazolium bromide, DmiBr.

NR, cis-1,4-polyisoprene (RSS1 type) was supplied by Torimex Chemicals (Lodz, Poland). It was characterized by a density of 0.930 g/cm^3^, with the contents as follows: volatile matter—0.56%wt., ash—0.33%wt., nitrogen—0.47%wt., and dirt—0.004%wt. A conventional curing system containing sulfur (Siarkopol, Tarnobrzeg, Poland) used as a curing agent was employed to vulcanize NR rubber compounds. A microsized zinc oxide (ZnO) demonstrated a specific surface area of 10 m^2^/g (Huta Bedzin, Bedzin, Poland) along with stearic acid (St. A.) (Sigma-Aldrich, Poznan, Poland) was applied to activate the vulcanization process, whereas 2-mercaptobenzothiazole (MBT) (Brenntag Polska, Kędzierzyn-Koźle, Poland) was used as an accelerator. Natural bio-waste such as ground chicken eggshells (ES) (Pokusa for Health, Sygneczów, Poland) was applied as a biofiller for NR compounds. Additionally, silanes, i.e., (3-aminopropyl)-triethoxysilane (APTES) and bis[3-(triethoxysilyl)propyl] tetrasulfide (TESPTS); ionic liquids, i.e., 1-butyl-3-methylimidazolium chloride (BmiCl) and 1-decyl-3-methylimidazolium bromide (DmiBr); and a surfactant, cetyltrimethylammonium bromide (CTAB), were employed to improve the dispersion degree of the biocomponent added in the elastomer matrix. All mentioned compounds were supplied by Sigma-Aldrich (Poznan, Poland). The structure of additives used is presented in Figure 1.

### 2.2. Characterization of Pure EGGSHELLS

Fourier transform infrared spectroscopy (FT-IR) absorbance spectra were recorded in the wavenumber range of 4000–400 cm^−1^ with 64 scans. Analysis was performed using a Thermo Scientific Nicolet 6700 FT-IR (Thermo Fisher Scientific, Waltham, MA, USA) spectrometer. The OMNIC 3.2 software was used to develop the spectra. The attenuated total reflectance (ATR) technique with a single reflection diamond ATR crystal was applied for investigations.

Thermogravimetry (TG) was employed to explore the thermal behavior of pure eggshells. Thermogravimetry/differential scanning calorimetry TGA/DSC1 analyzer (Mettler Toledo, Greifensee, Switzerland) was used to carry out measurements. The ground eggshell powder was heated in the temperature range of 25–1100 °C with a heating rate of 20 °C/min. The research was performed in an argon atmosphere with a gas flow of 50 mL/min.

### 2.3. Preparation and Characterization of Rubber Compounds

NR composites with the general formulations given in Table 1 and Table 2 were manufactured exploiting a laboratory two-roll mill (David Bridge & Co., Rochdale, UK) with the following roll dimensions: D = 200 mm, L = 450 mm. During compounding, the rotational speed of the front roll was 16 min^−1^, whereas the friction and the width of the gap between rollers were 1–1.2 mm and 1.5–3 mm, respectively. First, the unfilled rubber compounds were prepared as the reference and marked as unfilled sample, BmiCl, DmiBr, and CTAB. Then the compounds containing various amounts by weight of ES were manufactured (marked as 10ES, 20ES, 30ES, and 40ES). In the case of unfilled rubber compounds, each of them were prepared separately. Regarding the rubber compounds containing 40 phr of eggshells, the master batch was first manufactured, which contained rubber, ground eggshells, vulcanization accelerator MBT, eggshells, ZnO, and stearic acid. To ensure a sufficiently long mixing time allowing for homogeneous distribution of the filler without the risk of pre-vulcanization (scorching) of the rubber compounds during their preparation, sulfur was not added at this stage. Next, the master batch was weighed and cut into six pieces of equal weight. The sulfur with the appropriate additives, i.e., silanes, ionic liquids, or CTAB, were then introduced into each of them. The average temperature of the rolls during mixing was approximately 30 °C. The final rubber compounds were marked as follows: 40ES/APTES, 40ES/TESPTS, 40ES/CTAB, 40ES/BmiCl, 40ES/DmiBr.

The vulcanization process of rubber compounds was studied at 160 °C according to the ISO 6502 [38] standard procedures by employing the rotorless rheometer D-RPA 3000 produced by MonTech (Buchen, Germany). Based on the rheological measurements, the optimal vulcanization time (t_90_) and the scorch time (t_02_) were investigated. The t_90_ was determined as a parameter referring to the time required for the rubber compound to reach 90% of the maximum torque. On the other hand, the t_02_ was determined as the time at which the rubber compound reached 2% of the maximum torque achieved.

A differential scanning calorimeter DSC1 (Mettler Toledo, Greifensee, Switzerland) was adopted to establish the range of NR vulcanization temperatures and the enthalpy of vulcanization. Measurements were performed according to ISO 11357-1 [39] standard procedures. Small specimens of rubber compounds, with a mass of approximately 11 mg, were placed in an aluminum crucible. After cooling to –150 °C, the samples were heated to 250 °C in an argon atmosphere at a heating rate of 10 °C/min. Liquid nitrogen was used as a cooling agent.

The crosslink density was determined based on equilibrium swelling of the vulcanizates in toluene, according to the procedure described in ISO 1817 [40] standard. The Flory–Rehner equation [41] was adopted to calculate the crosslink density of vulcanizates. The Huggins parameter of NR-toluene interaction (*χ*) given by Equation (1) was used for calculations, where *V_r_* is the volume of the elastomer fraction in swollen gel [20].

(1) $$\chi =0.780+0.404V_{r},$$

Scanning electron microscopy (SEM) was employed to investigate the morphology and the dispersion degree of ground eggshells and other additives in the cured NR elastomer matrix. A LEO 1450 SEM microscope (Carl Zeiss AG, Oberkochen, Germany) was employed for taking SEM images of the vulcanizates and ground eggshell powder. Prior to the analysis, vulcanizates were broken down using liquid nitrogen, and then their fractures were covered with carbon. Samples of ground eggshells were also coated with carbon to improve the quality of the obtained SEM images.

The tensile properties of NR vulcanizates were explored using the ISO 37 [42] standard procedure. A Zwick Roell 1435 (Ulm, Germany) universal testing machine was adopted to perform measurements for five dumbbell-shaped samples of each vulcanizate with a thickness of approximately 2 mm and the width of the measuring section of 4 mm.

The Shore A hardness of NR vulcanizates was determined for disc-shaped specimens according to the standard procedures given in ISO 868 [43]. Zwick/Roell 3105 (Ulm, Germany) hardness tester was employed to perform measurements.

A DMA/SDTA861e analyzer (Mettler Toledo, Greifensee, Switzerland) was employed to perform dynamic mechanical analysis of the vulcanizates. Measurements were carried out in the tension mode. Cuboidal samples of the vulcanizates with a width of 4 mm, a length of 10.5 mm and a thickness of approximately 2 mm were used for the tests. First, the specimens were cooled to −150 °C and next heated to 80 °C with a heating rate of 3 °C /min. The measurements were carried out using a frequency of 1 Hz and a strain amplitude of 4 µm.

Following to the ISO 188 standard [44], the resistance to thermo-oxidative aging of the NR vulcanizates was examined. Plates of the vulcanizates with a thickness of approximately 2 mm were left in a drying chamber (Binder, Tuttlingen, Germany) at 70 °C for 10 days (240 h). Next, mechanical properties, crosslink density, and hardness of the vulcanizates were established and compared with the values obtained for non-aged vulcanizates. The aging coefficient (*A_f_*), which quantifies the aging resistance of material, was calculated according to Equation (2) [45], where *TS* is the tensile strength of vulcanizates, and *E_b_* is the elongation at break.

(2) $$A_{f}=\frac{{\left(E_{b}\times TS\right)}_{after\;aging}}{{\left(E_{b}\times TS\right)}_{befor\;eaging}},$$

Thermogravimetry (TG) was employed to explore the effect of ground eggshells and other additives on the thermal stability of NR vulcanizates. Thermogravimetry/differential scanning calorimetry TGA/DSC1 analyzer (Mettler Toledo, Greifensee, Switzerland) was used to perform two-step measurements. During the first segment of the measurement, specimens of vulcanizates were heated in the temperature range of 25–600 °C in an argon atmosphere using a gas flow of 50 mL/min. Next, the gas was changed into air (gas flow of 50 mL/min) and heating was continued up to 900 °C. A heating rate of 20 °C/min was used in both measurement segments.

The collected data were submitted to statistical analysis (one-way ANOVA) to compare the group means. Analysis of variance (ANOVA) was carried out to analyze the effect of the ground eggshells and tested additives on cure characteristics, crosslink density, mechanical properties, and thermal behavior of elastomer composites. The significance level was taken as P\0.05, i.e., for a level of confidence of 95%. Results of ANOVA analysis are given in the Supplementary Materials (Tables S1–S20).

## 3. Results and Discussion

### 3.1. Characterization of Pure Ground Eggshells

**Figure 2:**
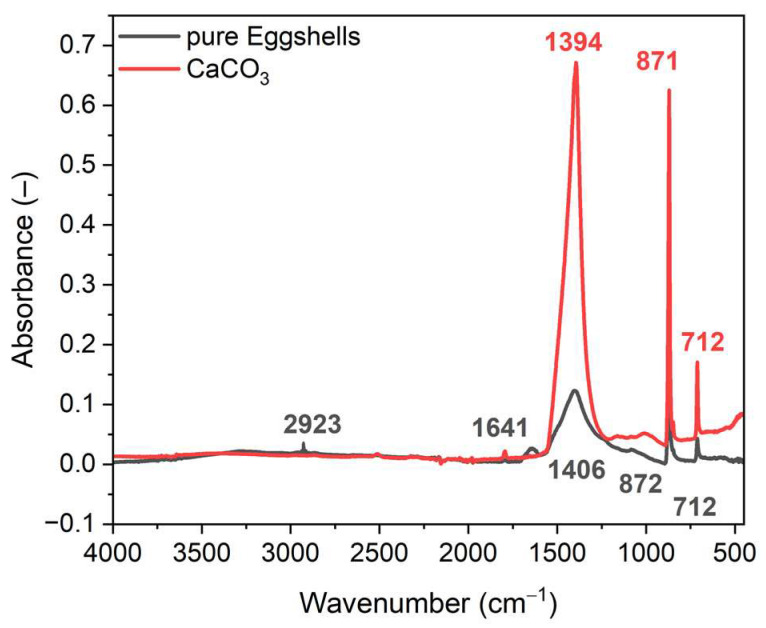
Fourier transform infrared spectroscopy (FITR) spectra for pure ground eggshells and calcium carbonate (CaCO_3_).

Fourier transform infrared spectroscopy (FTIR) was used to characterize the pure ground eggshells and to estimate their composition. Due to the heterogeneous structure of ground eggshells, prior to the FTIR measurement, a tablet was prepared by compressing the powder of ground eggshells under pressure. The same experiment was carried out for pure calcium carbonate (CaCO_3_) as the reference to compare its spectrum with that obtained for ground eggshells. The results are presented in Figure 2.

Analyzing the FTIR spectra of the pure eggshells and calcium carbonate presented in Figure 2, there were no meaningful differences in the position of the main FTIR bands. However, the intensity of the bands recorded for the pure eggshells was much lower compared to the CaCO_3_. According to the literature, the characteristic absorption bands of carbonate in CaCO_3_ occur at the wavenumbers of approximately 870 cm^−1^ (the out of plane bending vibrations), 1400 cm^−1^ (the asymmetric stretching vibrations), and 700 cm^−1^ (in plane bending vibrations) [46,47]. Consequently, FTIR spectroscopy confirmed that CaCO_3_ is the main component of ground eggshells due to the presence and overlap of bands characteristic for this compound in the FTIR spectrum recorded for eggshells. Characteristic bands for chalk in ground eggshells were identified at: 1406, 872, and 712 cm^−1^, respectively. Additional bands in the FTIR spectrum at a wavenumber of approximately 2923 cm^−1^ corresponded to the asymmetric and symmetric stretching vibrations of C-H and -CH_2_- groups, respectively. It resulted from the presence of organic components in the ground eggshells [48,49]. Moreover, the band at 1641 cm^−1^ was identified, which corresponds to the C=O stretching vibrations. Boronat et al. [50] reported that the presence of the organic components in eggshells could provide a good interaction between the biofiller and the elastomer, promoting the homogenous dispersion of eggshells in the elastomer matrix.

**Figure 3:**
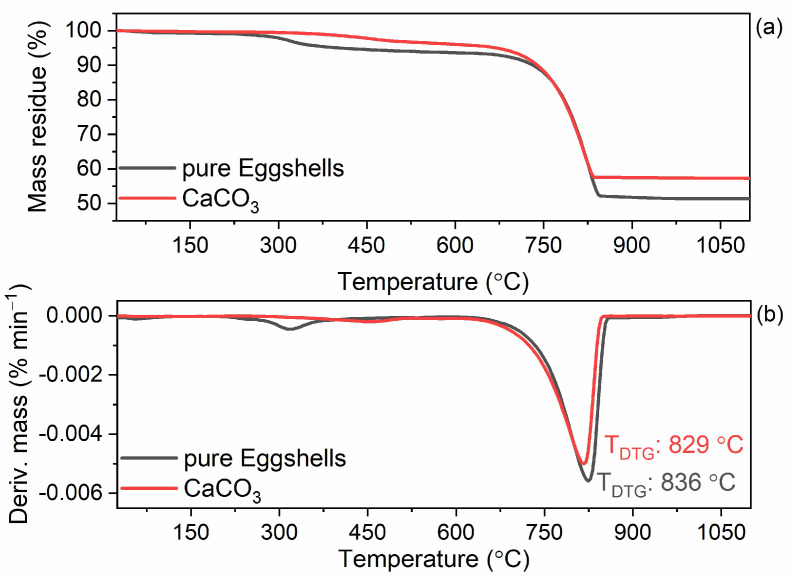
Thermogravimetric (TG) and derivative thermogravimetric (DTG) curves of the ground eggshells and CaCO_3_. (**a**) TG curves; (**b**) DTG curves.

In the next step, thermogravimetry (TG) was performed to study the thermal stability of the ground eggshells and to estimate their composition. The same measurement was performed for CaCO_3_ for comparison. The onset decomposition temperature (T_5%_) was determined as the temperature at which 5% of the mass loss occurred compared to the initial mass of the sample. Thermogravimetric (TG) and derivative thermogravimetric (DTG) curves for pure ground eggshells are plotted in Figure 3 and results are summarized in Table 3.

Analyzing the TG/DTG curves presented in Figure 3, it was observed that the thermal decomposition of ground eggshells and CaCO_3_ proceeded similarly, although some differences can also be noticed. Regarding the eggshells, in the temperature range of 200–450 °C, a slight mass loss of approximately 6.3% occurred, which was assigned to the thermal decomposition of the organic compounds contained in eggshells (Table 3) [47,49,51]. Due to the presence of organic compounds, the thermal stability of ground eggshells was poorer compared to pure CaCO_3_. Thus, the T_5%_ of eggshells was of approximately 403 °C, whereas T_5%_ of CaCO_3_ was 667 °C, respectively. The main degradation process of ground eggshells proceeded with a T_DTG_ of 836 °C and a mass loss of approximately 42.3%, so similar to the thermal decomposition of pure CaCO_3_ (T_DTG_ of 829 °C, mass loss of approximately 38.9%). In the case of CaCO_3_, the mass loss in the temperature range of 650–900 °C was attributed to the emission of gaseous carbon dioxide (CO_2_) [49,52,53]. The presence of a similar mass loss on the TG curve of eggshells in the temperature range of 650–900 °C confirmed that the eggshells contained CaCO_3_.The removal of CO_2_ from CaCO_3_ led to the calcium oxide (CaO) as a mineral residue after thermal decomposition [54,55,56]. The residue after decomposition at 1100 °C was 51.4% for eggshells and 57.2% for CaCO_3_, respectively. Therefore, the ground eggshells contained of approximately 94% mineral compounds, i.e., CaCO_3_, whereas the organic part was approximately 6% mineral compounds, which is consistent with the literature [49,56].

### 3.2. Cure Characteristics and Crosslink Density of NR Composites Filled with Eggshells

The influence of the amount of ground eggshells and the additives used to improve their dispersion in the elastomer matrix on the rheometric properties and crosslink density of NR composites was established. Rheometric measurements were performed at 160 °C, and the results are given in Table 4.

The minimum torque (S_min_) during rheometric measurement refers to the viscosity of the uncured rubber compound, which is an important parameter of their processing, especially by extrusion or injection molding. The value of S_min_ determined for the unfilled NR compound was 0.4 dNm. Considering the measurement error, the amount of eggshells and applied additives, such as CTAB, silanes, and ionic liquids (ILs), did not significantly affect the S_min_ and therefore the viscosity of uncured NR biocomposites (Table 4), which is important from a technological view point.

On the other hand, the rheometric torque increment (∆S) results from the increased stiffness of the rubber composite due to vulcanization and thus, it can be referred to the crosslinking degree of elastomer [57]. Regarding the unfilled NR compounds, ILs and CTAB increased the ∆S from 5.1 dNm to approximately 7 dNm due to the increased crosslinking degree of the elastomer. It was confirmed by the values of the crosslink density, which for the unfilled vulcanizates containing ILs and CTAB were significantly higher compared to the benchmark without these additives. It should be noticed crosslinking reactions take place at the interface between the components of the crosslinking system and the rubber, whereas quaternary ammonium salts and ILs can catalyze the interphase reactions, probably resulting in the increased crosslink density of the vulcanizates. Moreover, CTAB and ILs were reported to improve the dispersion degree of the crosslinking system components in the elastomer matrix, thus improving the contact between the curatives and consequently the crosslink density of the vulcanizates [58,59,60]. Moreover, when filler is applied, the polymer/filler interactions lead to a stiffening of the internal structure of the polymer material, which is determined by the hydrodynamic effect of the filler [61]. Rubber compounds filled with eggshells exhibited higher ∆S compared to the unfilled benchmark. Moreover, ∆S of the eggshell-filled NR compounds increased with the content of the filler. Similar to the unfilled composites, introducing additives, especially CTAB and ILs, additionally increased this parameter compared to the unfilled sample and rubber compound without the additives (40ES). It probably resulted from the improved elastomer/biofiller compatibility after application of those additives due to their widely known positive impact on the dispersion degree of rubber compounds ingredients in the elastomer matrix and crosslinking degree of the elastomer. Therefore, vulcanizates with CTAB and ILs should exhibit higher crosslink density than those without them.

Regardless of their amount, application of ground eggshells had no impact on the scorch time (t_02_) and thus the processing safety of the of rubber composites at 160 °C. Silanes, the surfactant CTAB, and ILs did not affect the t_02_ of NR compounds either.

The degree of crosslinking of rubber compounds affects the properties of the final rubber products. Therefore, the optimal vulcanization time (t_90_) is a very important parameter which ensures the production of final products having required properties [62]. Moreover, shorter vulcanization times are more desirable due to the reduction of the total cost of rubber compounds processing. The unfilled benchmark exhibited the t_90_ of 2 min. Application of ground eggshells and additives, such as ILs and silane APTES, did not have a significant influence on the t_90_ of NR compounds compared to the unfilled benchmark. Only rubber compounds with CTAB and silane TESPTS exhibited slightly longer t_90_ of approximately 3 min.

The crosslink density (ν_t_) of NR vulcanizates filled with eggshells was slightly improved in comparison with that of the unfilled sample (Table 4), especially for 30 phr and 40 phr of eggshells, which confirmed the beneficial impact of this biofiller on the vulcanization of NR compounds. Moreover, the ν_t_ of the NR vulcanizates slightly increased with the amount of ground eggshells introduced to the elastomer. Thus, application of ground eggshells as biofiller for NR composites, alternatively to other non-reinforcing fillers, i.e., talc or chalk [63], seems to be reasonable since vulcanizates filled with eggshells demonstrated improved crosslink density compared to the unfilled sample. Silanes, i.e., APTES and TESPTS, did not significantly affect the ν_t_ of NR vulcanizates filled with eggshells. As expected CTAB, BmiCl, and DmiBr increased the ν_t_, which probably resulted from their positive influence on the dispersion of biofiller and curing system components in the elastomer matrix. CTAB is commonly used for improving the filler–elastomer interaction [32,64]. Furthermore, CTAB as a surfactant may be preferentially adsorbed on the surface of the filler particles. As a result, the ability of filler to adsorb the curatives is reduced, therefore enhancing the crosslinking efficiency and consequently the crosslink density of the vulcanizates. A similar effect has been described for ILs, which can boost the vulcanization and consequently improve the curing characteristics and crosslink density of elastomers [27,28,65].

### 3.3. Differential Scanning Calorimetry (DSC) Analysis of the Crosslinking Process of NR Compounds

**Figure 4:**
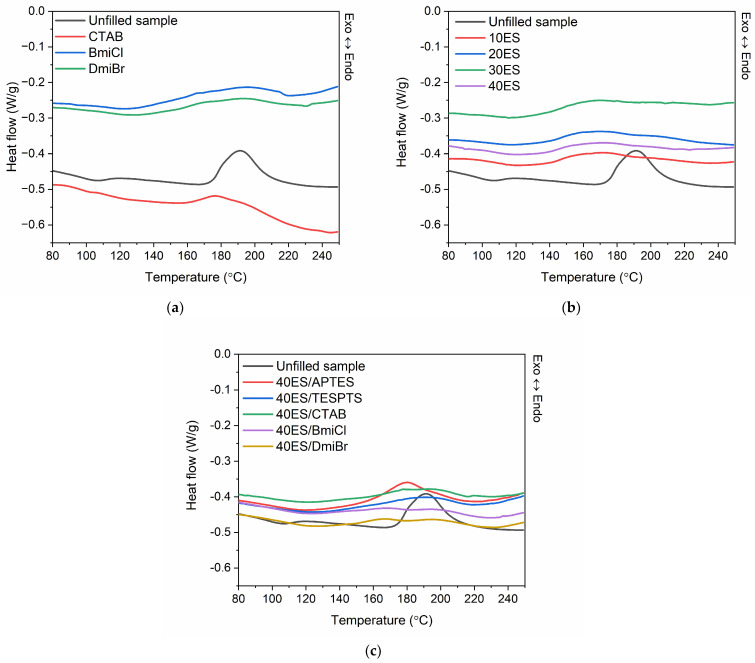
DSC curves for vulcanization of NR compounds: (**a**) without filler; (**b**) with addition of eggshells; (**c**) with addition of eggshells, silanes, CTAB, and ILs.

DSC analysis was employed to determine the influence of ground eggshells and other additives, i.e., silanes, CTAB, and ILs, on the vulcanization temperature and the enthalpy of this process. The results of DSC analysis are summarized in Table 5, whereas DSC curves of NR composites filled with eggshells are plotted in Figure 4.

Vulcanization is an exothermic process which can be identified as a peak on DSC curve and that can be integrated by determining the amount of heat released in this process. Regarding DSC curve of the unfilled NR compound, it was observed that the vulcanization proceeded in a temperature range of 174–210 °C (Figure 4). After starting at about 174 °C, vulcanization proceeded intensively with the enthalpy of approximately 13.8 J/g. Applying CTAB and ILs in the unfilled NR compounds lowered the onset vulcanization temperature by approximately 30 °C and so had a significant influence on this process. Thus, DSC results confirmed the beneficial, catalytic impact of these additives on the vulcanization, which was concluded based on the rheometric data. Furthermore, DSC analysis revealed that the content of ground eggshells in NR compounds affected the vulcanization temperature but no simple correlation between the amount of eggshells and the enthalpy of vulcanization was observed. NR compounds filled with ground eggshells demonstrated by 36–38 °C lower onset vulcanization temperatures and by 2–6 J/g lower ∆H_vul_ compared to the unfilled NR. Thus, the vulcanization of rubber compounds containing biofiller started at a lower temperature and was accompanied by the release of less heat (especially in the case of 40ES rubber compound) compared to the unfilled NR. Silanes, i.e., APTES and TESPTS, increased the onset vulcanization temperature by 15–20 °C compared to 40ES. Moreover, APTES significantly raised the energetic effect of vulcanization compared to rubber compounds with other additives, which proved that it can be used as substance promoting crosslinking reactions [66]. ILs and CTAB did not significantly affect the onset vulcanization temperature and enthalpy of vulcanization compared to 40ES, but it should be noticed that eggshell-filled rubber compounds with these additives exhibited significantly lower onset vulcanization temperature then the unfilled NR. Most importantly, DSC analysis, together with rheometric measurements, confirmed that ground eggshells had no detrimental influence on the course and efficiency of NR vulcanization, whereas CTAB and ILs can be successfully used to reduce the vulcanization temperature and increase the crosslink degree of the NR composites.

### 3.4. Dispersion of Ground Eggshells and Curatives in NR Composites

**Figure 5:**
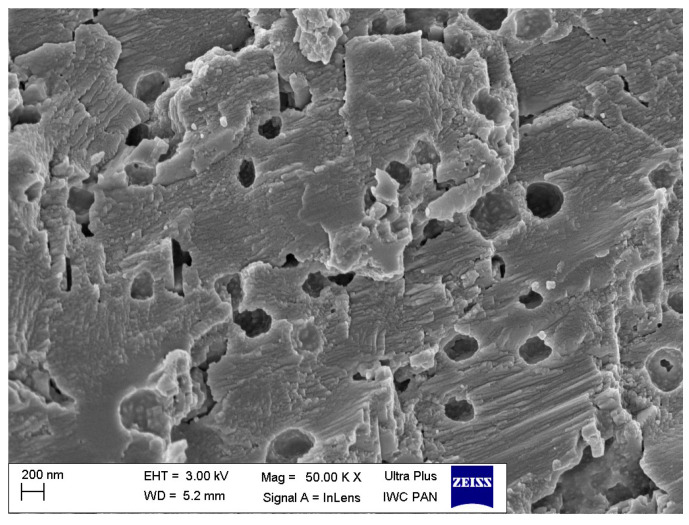
Scanning electron microscopy (SEM) image of the pure ground eggshells.

**Figure 6:**
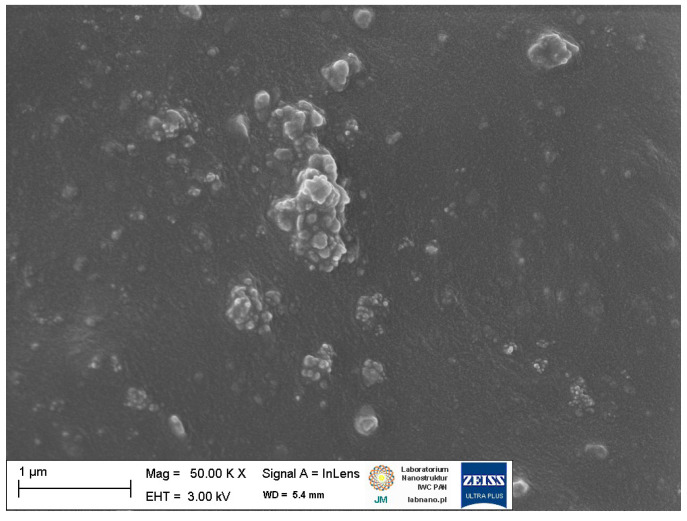
SEM image of the unfilled NR vulcanizate.

**Figure 7:**
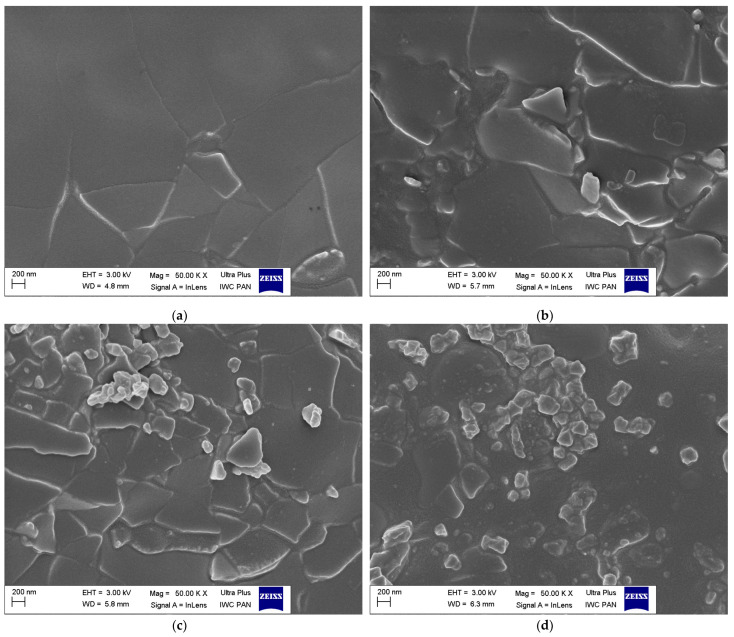
SEM images of NR vulcanizates filled with different amounts of ground eggshells: (**a**) 10ES; (**b**) 20ES; (**c**) 30 ES; (**d**) 40ES.

**Figure 8:**
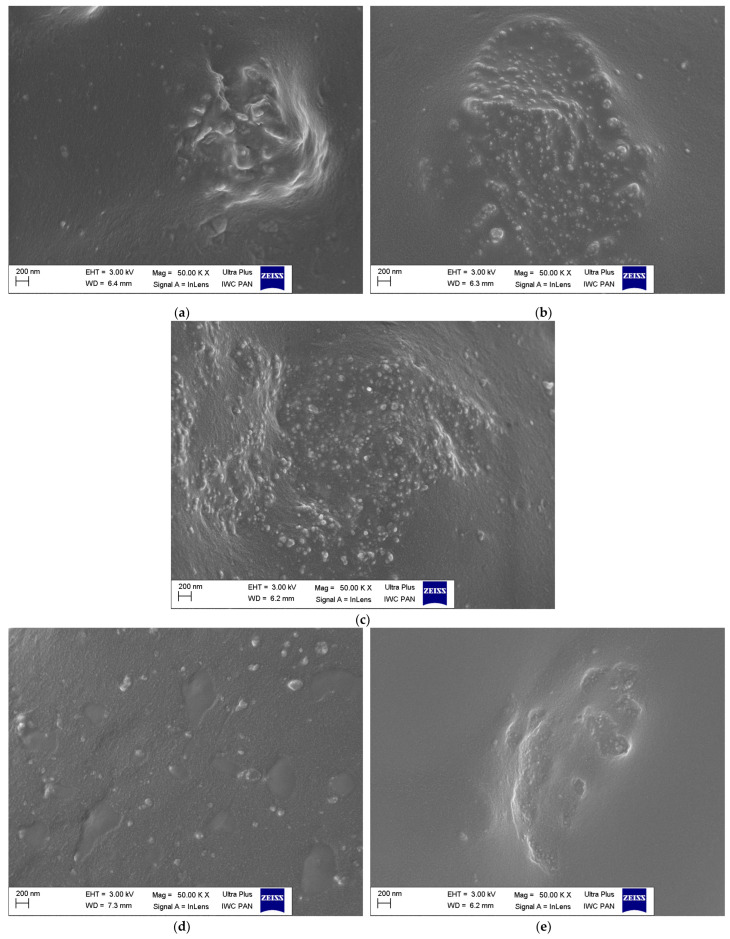
SEM images of NR vulcanizates filled with 40 phr of ground eggshells containing additives: (**a**) APTES; (**b**) TESPTS; (**c**) CTAB; (**d**) BmiCl; (**e**) DmiBr.

Scanning electron microscopy (SEM) images of pure ground eggshell powder was taken to study the morphology and size of biofiller particles. One of the most crucial issues in the elastomer technology is the uniform dispersion of the filler particles. The structure and size of the filler particles and the degree of filling affect the interfacial interactions and the morphology of the obtained composite and thus the functional properties of the final rubber product [2]. Furthermore, the interaction between the curatives and the elastomer should be intensified for enhancing the crosslinking efficiency. It has also been confirmed that some additives exhibit plasticizing effects and consequently improve the dispersion of fillers in the elastomer matrix [67,68,69]. Therefore, the effect of silanes, CTAB, and ILs on the dispersion of ground eggshells and curatives in the elastomer matrix was investigated using SEM. Results obtained for the pure eggshell powder and for the unfilled sample are presented in Figure 5 and Figure 6, respectively. SEM images of NR vulcanizates filled with ground eggshells are given in Figure 7 and Figure 8.

Due to the mechanical grinding process, the morphology of pure ground eggshell particles presented in Figure 5 is irregular. The particles have a platelet structure, angular/ sharp edges, and a rough surface. Moreover, the porous structure of their surface is observed. It can be supposed that these various pores and cavities could provide larger exposition surface area for the adsorption of the curing system and/or additives used in this research, i.e., CTAB, ILs, or silanes. The particles of ground eggshells are very heterogenous in size, and their particle size is rather in the micrometer range. A relatively thin layer of eggshells, which is formed by spherulitic aggregates of crystals, merges into a compact layer [70,71,72]. It can be assumed that a filler with such morphology and particle structure will be difficult to disperse in the elastomer matrix.

SEM image of the unfilled NR vulcanizate (Figure 6) showed quite homogenous dispersion of the curing system in the elastomer matrix. However, single small agglomerates with a size of much less than 1 µm can be seen. These agglomerates are embedded in the elastomer film. Regarding NR composites containing ground eggshells, SEM images for 10, 20, 30, and 40 phr of eggshells are presented in Figure 7a–d.

SEM analysis confirmed that increasing in ground eggshell content in the NR composites from 10 phr to 40 phr affected the dispersion of biofiller and the curing system in the elastomer matrix. The ground eggshell particles are dispersed in the elastomeric matrix in the form of angular plates of irregular shape and size. Near the micrometric filler plates, especially in the case of 30 phr and 40 phr of the filler, clusters of particles with a nanometer size are visible, which may belong to the curing system. It can also be finer particles of eggshells that have been broken up by the shear forces applied during the preparation of the elastomer compound using a rolling mill.

SEM analysis of NR vulcanizates filled with 40 phr of ground eggshells containing silanes, CTAB, and ILs (Figure 8a–e) showed that these additives had a significant influence on the dispersion of biofiller in the elastomer matrix, including the size and the structure of agglomerates. Composites containing APTES and TESPTS exhibited more homogenous dispersion of ground eggshells and curatives compared to the vulcanizate without these additives (Figure 8a,b). The SEM images show agglomerates having a size of approximately several micrometers, which are clusters of particles with a size below 200 nm. These agglomerates are embedded with an elastomer film. The lamellar morphology of the filler particles, which was observed for the silane-free vulcanizate, is not visible. CTAB had a similar effect on the dispersion of ground eggshells in NR elastomer to silanes (Figure 8c). Generally, CTAB, similar to silanes, is known to improve the solubility and the dispersion of solid particles in elastomers [73,74]. The most homogeneous dispersion of the biofiller and curing system was provided by ILs, especially BmiCl (Figure 8d,e). The beneficial effect of ILs on the dispersion of solid components in the elastomer composites was confirmed in our previous work [29,66,75]. On the other hand, Haddad et al. [76] confirmed that eggshells were able to interact with imidazolium ILs, which influenced the crystal structure of eggshells. Such interactions may facilitate the homogeneous dispersion of the ground eggshells in the elastomer matrix. Moreover, other researchers also reported that ILs, including BmiCl, could act as coupling agents due to their ability to interact directly with both hydrophobic elastomer matrices and hydrophilic fillers [77,78].

### 3.5. Tensile Properties and Hardness of NR Composites Filled with Eggshells

It is commonly known that elastomer properties are strongly dependent on the crosslink density [79] and dispersion of the filler particles in the elastomer matrix [65]. Therefore, the influence of ground eggshells on the tensile properties and hardness of the NR vulcanizates containing various additives was explored, and the results are collected in Table 6.

The data collected in Table 6 confirmed that mechanical properties of NR vulcanizates depended on the amount of the ground eggshells. Regarding the stress at a relative elongation of 300% (SE_300_), the unfilled benchmark showed the SE_300_ value of 1.2 MPa. Vulcanizates filled with eggshells demonstrated slightly higher SE_300_ compared to the unfilled benchmark. Applying the silane APTES, ILs, and CTAB resulted in the further increase in the SE_300_ compared to the unfilled NR and 40ES vulcanizate. It resulted from the higher crosslink density of the vulcanizates containing these additives compared to unfilled sample and 40ES. It is commonly known that SE_300_ is strongly dependent on the crosslink density of vulcanizates and increases with ν_t_ [80].

Analyzing the tensile strength (TS) and elongation at break (E_b_), the unfilled vulcanizate reached the TS value of 9.6 MPa and EB of 820%. The amount of eggshells slightly affected the TS of vulcanizates. The highest TS of approximately 11 MPa was demonstrated by the vulcanizate containing 30 phr of the biofiller. Vulcanizates with silanes exhibited lower TS than the unfilled composite and 40ES vulcanizate without silanes. On the other hand, CTAB and ILs, especially DmiBr, improved the TS compared to the unfilled benchmark and vulcanizate filled with 40 phr of ground eggshells. It should be noted that CTAB and ILs had the most pronounced and the most beneficial influence on the dispersion of ground eggshells in the elastomer matrix. Moreover, vulcanizates with CTAB and ILs showed the highest crosslink densities. Thus, the homogeneous dispersion of the filler particles and enhanced crosslink density of the vulcanizates containing CTAB and ILs resulted in the TS improvement.

Regarding the elongation at break (E_b_), introducing ground eggshells to NR composites had a significant effect on the elasticity of the vulcanizates since E_b_ was reduced by approximately 150% compared to the unfilled benchmark. It resulted from the increased stiffness of the vulcanizates due to both the addition of the ground eggshells as a filler and increased crosslink density compared to the unfilled NR. Moreover, vulcanizate containing CTAB had the lowest E_b_ of 569%, which was due to the highest crosslink density of this vulcanizate.

Not only E_b_ but also hardness strongly depends on the filler’s addition and crosslink density of vulcanizates. As expected, the unfilled benchmark demonstrated the lowest hardness of approximately 31 Shore A. The amount of eggshells introduced to NR composites affected their hardness. The hardness increased by 3–7 Shore A with the loading of eggshells in the rubber composite. Addition of CTAB and ILs caused an additional increase in the hardness by approximately 4–6 Shore A compared to the 40ES vulcanizate, which did not contain these additives. It was a consequence of the higher crosslink density of the vulcanizates containing CTAB and ILs. Since silanes had no significant impact on the crosslink density compared to vulcanizate filled with 40 phr of eggshells, they did not significantly affect the hardness of the vulcanizates.

The reinforcing effect of eggshells was demonstrated by slight increase in the tensile strength, SE_300_ and hardness of the vulcanizates, with reduced elongation at break compared to the unfilled composite. Taking into account the effect of eggshells on the discussed parameters, this biofiller can be regarded as an inactive filler, which can be used as an alternative to commercial non-reinforcing fillers, such as talk or chalk [63,81].

### 3.6. Dynamic Mechanical Properties of NR Composites Filled with Eggshells

**Figure 9:**
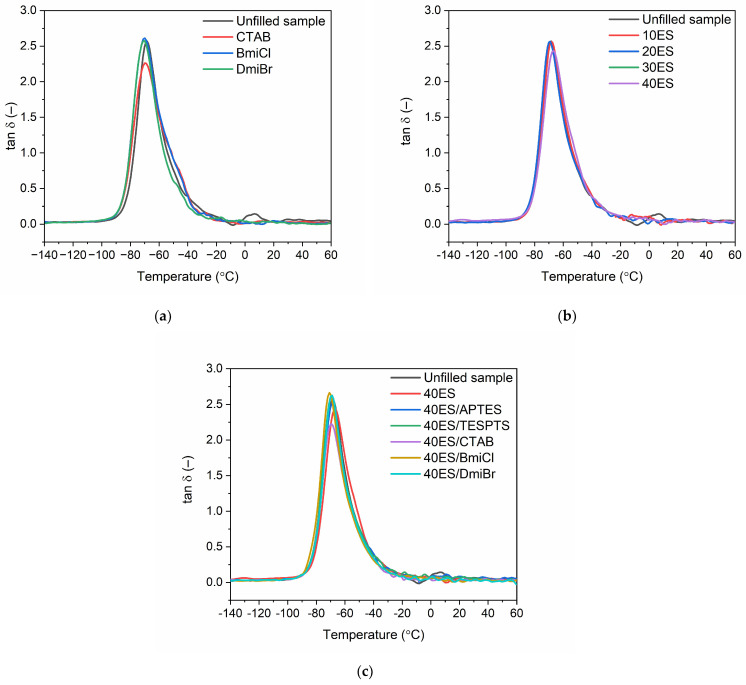
Loss factor (tan δ) curves versus temperature for NR vulcanizates: (**a**) without filler; (**b**) with addition of eggshells; (**c**) with addition of eggshells, silanes, CTAB, and ILs.

Dynamic mechanical analysis (DMA) was used to study the influence of ground eggshells and tested additives on the viscoelastic properties of NR composites and their ability to dampen vibrations. The analysis was conducted as a function of temperature. The results are summarized in Table 7, whereas the DMA curves of NR vulcanizates filled with eggshells are plotted in Figure 9.

The glass transition temperature corresponds to the maximum of the tan δ peak. DMA curves of NR vulcanizates filled with ground eggshells plotted in Figure 9 showed the glass transition of cured NR in the temperature range from −90 °C to −40 °C. It was confirmed by the presence of the peak of mechanical loss factor (tan δ) on the DMA curves. The glass transition temperature (T_g_) of NR determined for the unfilled vulcanizate was −68 °C. Applying CTAB and ILs did not influence the T_g_ of the unfilled NR, whereas the T_g_ of the vulcanizates filled with eggshells was by approximately 1–2 °C lower. Therefore, the amount of eggshells as well as the additives had no meaningful impact on the T_g_ of NR composites, which was within the standard deviation of the obtained data.

Tan δ is commonly used as a measure of the material’s ability to dampen vibrations. The unfilled vulcanizate reached the highest value of tan δ at T_g,_, which was of approximately 2.7 (Table 7). It was a consequence of the highest flexibility of this vulcanizate and the highest mobility of the elastomer chains in the unfilled compound. The addition of ground eggshells and other additives, i.e., silanes, CTAB, and ILs, had no remarkable effect on the tan δ at T_g_ compared to the unfilled NR sample. However, both vulcanizates containing CTAB, i.e., the unfilled one and that filled with 40 phr of eggshells, were characterized by the lowest tan δ at T_g_, which was of approximately 2.3 (Figure 9b). This could result from the highest crosslink density of these vulcanizates compared to other NR composites.

Regarding the values of tan δ at 25 °C and 50 °C, which were determined in the rubbery elastic region (Table 7), the addition of eggshells and other additives did not considerably affect the discussed parameter compared to the unfilled benchmark. NR vulcanizates filled with eggshells were characterized with a good vibration damping ability and exhibited stable properties in dynamic conditions.

### 3.7. Thermo-Oxidative Aging Resistance of NR Vulcanizates Filled with Eggshells

**Figure 10:**
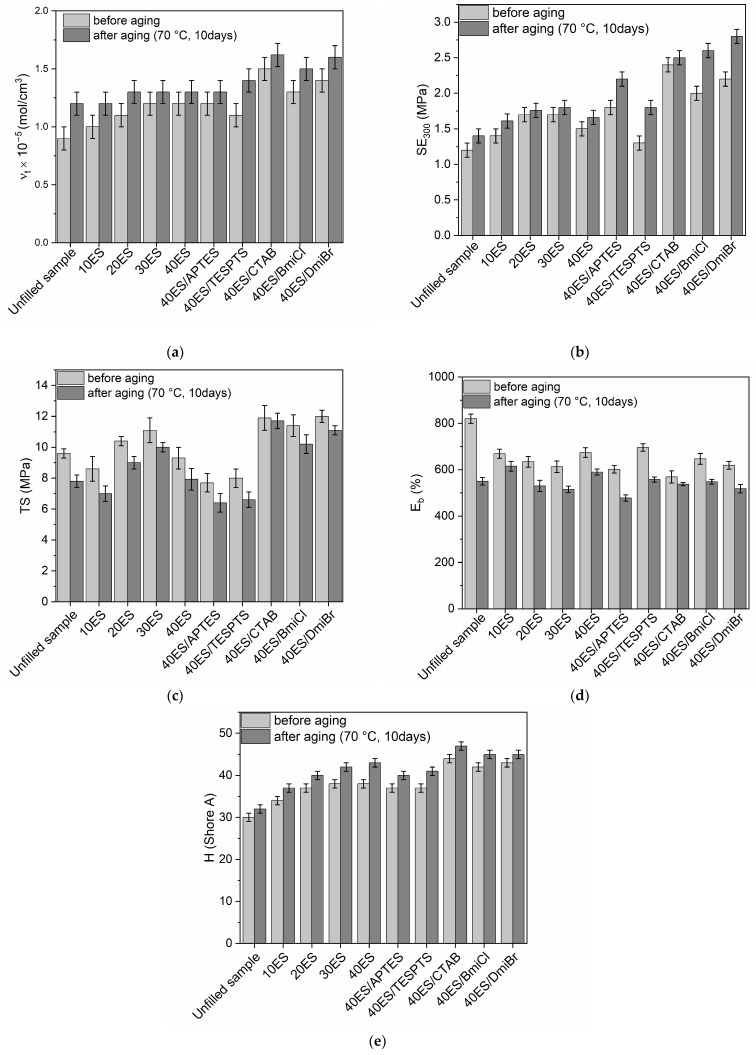
Influence of thermo-oxidative aging on the properties of NR vulcanizates filled with eggshells: (**a**) crosslink density; (**b**) stress at 300% relative elongation; (**c**) tensile strength; (**d**) elongation at break; (**e**) hardness.

One of the disadvantages of natural rubber is poor resistance to thermo-oxidative aging compared to most synthetic elastomers [82,83]. Therefore, if various additives are to be used, they should not worsen the aging resistance. Therefore, the influence of eggshells, silanes, ILs, and CTAB on the resistance of vulcanizates to thermo-oxidative aging was explored based on the changes in the mechanical properties and crosslink density. The study was performed in accordance with the methodology provided in our previous works [28,66]. The results are presented in Figure 10.

As expected, vulcanizates filled with ground eggshells and containing additives exhibited higher crosslink densities after exposure to 70 °C for 10 days (Figure 10a). The same effect of thermo-oxidative aging on the crosslink density was observed for unfilled vulcanizate. Thus, further crosslinking of NR composites occurred under prolonged exposure to elevated temperature. This could be due to the dissociation of the existing sulfur crosslinks in the elastomer network and thus the formation of new crosslinks by free sulfur, as reported by Choi et al. [84]. Similar effect of prolonged thermo-oxidation on the crosslink density was also reported by other researchers [85,86].

The influence of thermo-oxidation on the SE_300_ of NR vulcanizates is shown in Figure 10b. SE_300_ is strongly dependent on the ν_t_ of the vulcanizates. Thus, the impact of thermo-oxidative aging on the SE_300_ correlated with the changes in the ν_t_. Consequently, vulcanizates demonstrated higher SE_300_ after thermo-oxidative aging than non-aged samples.

As expected, prolonged exposure to elevated temperature reduced the TS of both the unfilled benchmark and the vulcanizates filled with ground eggshells (Figure 10c). Similar effect of thermo-oxidative aging on the TS was noticed for the vulcanizates containing silanes, CTAB and ILs. It was due to further crosslinking of the elastomer during aging, which caused the vulcanizates to be over-crosslinked and thus poorly resistant to mechanical stress [87,88]. The content of the biofiller did not remarkably affect the changes in TS after prolonged thermo-oxidation.

As a consequence of thermo-oxidative aging, the E_b_ of NR vulcanizates was significantly lower compared to non-aged samples (Figure 10d). The greatest decrease in E_b_ due to the aging process, by approximately 270%, was recorded for the unfilled sample. It was due to the increase in the crosslink density upon thermo-oxidation. Vulcanizates filled with ground eggshells as well as containing CTAB, silanes, and ILs were also characterized by lower E_b_ compared to non-aged samples. However, the changes in E_b_ of these vulcanizates were less significant than for the unfilled elastomer.

The thermo-oxidative aging had a slight effect on the hardness of NR vulcanizates. Hardness after aging was by approximately 2 Shore A higher compared to that of non-aged samples (Figure 10e).

Having studied the influence of thermo-oxidative aging on the tensile properties of NR vulcanizates filled with eggshells, we then determined their aging factor (A_f_) using the changes of TS and E_b_ of vulcanizates upon the aging process. The results are given in Table 8.

When the value of A_f_ is close to 1, the aging process does not considerably affect the tensile properties of the vulcanizates, and, consequently, the material shows good resistance to thermo-oxidative aging. The unfilled NR vulcanizate exhibited the A_f_ of approximately 0.5, and thus it showed rather poor resistance to thermo-oxidative aging (Table 8). It was due to the structure of NR elastomer, i.e., the presence of double bonds, which are active centers for oxidation reactions resulting in the degradation of polymer [89]. The application of ground eggshells as a biofiller improved the resistance of NR vulcanizates to thermo-oxidative aging since the A_f_ of the vulcanizates increased to 0.7. The application of silanes had no impact on the resistance to thermo-oxidative aging compared to 40ES vulcanizate. On the other hand, NR vulcanizates containing CTAB and ILs showed significantly better resistance to prolonged thermo-oxidation compared to the unfilled NR and 40ES vulcanizate. The A_f_ of vulcanizates containing CTAB was of approximately 0.9, whereas for vulcanizates containing IL A_f_ was 0.8.

Most importantly, applying ground eggshells and the tested additives improved the resistance to thermo-oxidative aging, which is very important for technological applications of NR composites. This is an additional benefit of using eggshell waste as a biofiller for elastomers.

### 3.8. Thermal Stability of NR Vulcanizates Filled with Eggshells

**Figure 11:**
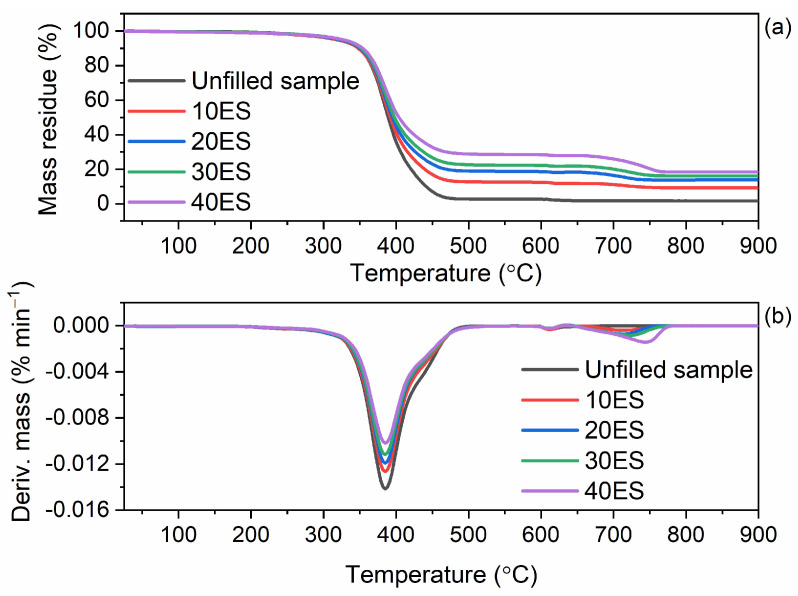
TG and DTG curves of the NR vulcanizates containing different amount of ground eggshells: (**a**) TG curves; (**b**) DTG curves.

**Figure 12:**
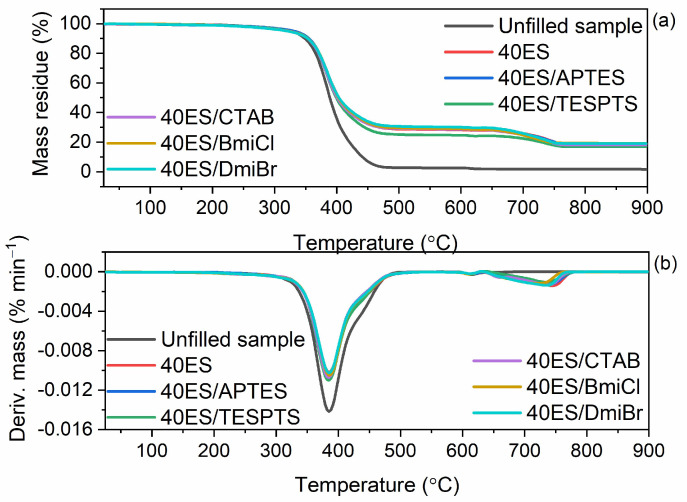
TG and DTG curves of NR vulcanizates filled with 40 phr of ground eggshells containing silanes, ILs, and CTAB: (**a**) TG curves; (**b**) DTG curves.

The elastomer matrix as well as applied additives, especially the organic ones, have an effect on the thermal stability of vulcanizates. Therefore, having established the thermal stability of pure ground eggshells, we then examined their effect on the thermal decomposition of NR composites. The results of TG analysis are summarized in Table 9, whereas TG and DTG curves are plotted in Figure 11 and Figure 12.

The results of TG analysis presented in Table 9, showed that ground eggshells and their amount did not significantly affect the onset decomposition temperature (T_5%_) of the NR vulcanizates. Thermal decomposition of the unfilled sample started at a temperature of 322 °C, and the main degradation process occurred at 398 °C with a mass loss of 97.1%. Introducing 10 phr of eggshells reduced the T_5%_ by approximately 3 °C compared to the unfilled benchmark. However, the T_5%_ increased with increasing the amount of biofiller in the vulcanizate, and for the vulcanizate filled with 40 phr of eggshells, T_5%_ was 328 °C, so 6 °C higher compared to the unfilled NR. Furthermore, ground eggshells had no significant impact on the T_DTG_, which was determined as the temperature of the maximum rate of the vulcanizates mass loss. T_DTG_ of the studied vulcanizates ranged from 395 °C to 398 °C. Exposure to elevated temperature results in several changes in physical or chemical composition of biocomposites which can lead to their degradation. Improving thermal degradation is equal with improving thermal stability of the biocomposite. This property is undoubtedly affected by such parameters as lower % of mass loss, higher onset decomposition temperature, lower rate of degradation, and the decomposition curve shifting up [90]. Several studies [91,92,93] showed that the onset decomposition temperature of biocomposite is lowered with the addition of natural fibers, which can be due to the fact that natural fibers have lower thermal stability that seemed to increase the deformation of the crystalline structure of additives at higher temperature [93]. There is no clear definition of what can improve the thermal stability of the material since it is an individual matter. However, it is known that the treatment of the fibers, additives, or raw materials from biomass can affect the compatibility of the fibers and the polymers of the biocomposites, that improve the resistance to degradation of biocomposites. Various types of modifications and processing of materials, which improved the thermal stability, were discussed in several works [19,22,64].

The additives used to improve the dispersion of the biofiller in the elastomer matrix did not significantly affect either the T_5%_ or T_DTG_ compared to the unfilled NR and 40ES vulcanizate. The T_5%_ of NR vulcanizates containing additives was in the range of 322–331 °C, whereas T_DTG_ of most vulcanizates was of approximately 395 °C. The most thermally stable were the vulcanizates containing silane APTES. As expected, application of the biofiller affected the mass loss during thermal decomposition of the vulcanizates. The mass loss at 25–600 °C for the eggshells-filled vulcanizates was much lower compared to the unfilled benchmark and decreased when the amount of the biofiller increased. The lower mass losses at 25–600 °C for the vulcanizates with biofiller resulted from the lower rubber content in the same amount of rubber composite compared to the unfilled NR. Applied additives had no meaningful impact on the mass loss at 25–600 °C compared to 40ES. On the other hand, the mass loss at 600–900 °C was much higher for the vulcanizates containing biofiller and increased with the increase in the content of ground eggshells. It was due to the thermal decomposition of the CaCO_3_ contained in eggshells, which in this temperature range decomposed with the release of CO_2_. Therefore, the higher the content of CaCO_3_ in the composite, the more CO_2_ was released during the vulcanizate decomposition. Applying silanes, CTAB, and ILs did not significantly affect the mass loss at 600–900 °C. The residue at 1100 °C was significantly higher for the vulcanizates filled with ground eggshells compared to the unfilled benchmark and increased with the content of the biofiller in the vulcanizate. In the case of unfilled NR, the residue at 900 °C consisted of ash and zinc oxide, which was used as vulcanization activator. The residue at 900 °C of the eggshells-filled vulcanizates contained additionally CaO, which resulted from the thermal decomposition of CaCO_3_. Hence, the residue at 900 °C was the greater the higher the content of eggshells in the vulcanizate. Most importantly, ground eggshells and applied additives did not worsen the thermal stability compared to the unfilled vulcanizate. NR composites filled with ground eggshells were characterized by thermal stability up to a temperature of approximately 320–330 °C. Thus, they were characterized by appropriate thermal stability.

To summarize, in general it is well known that biocomposites consisting of natural fibers, biomass, and biopolymers have the environmental benefits of being renewable, biodegradable and sustainable. However, it is worth remembering that they also have several disadvantages, such as poor mechanical properties, low thermal stability, and high water absorption. Usually, they are characterized by poor resistance to aging processes. However, the level of these disadvantages is individual and varies from one biocomposite to another due to there being lots of factors that determine these properties. To clarify, referring to the composites with most used fillers, i.e., silica, tensile strength of NR vulcanizates was of approximately 8–12 MPa [65]. Moreover, NR vulcanizates filled with silica were characterized with improved thermal stability compared to the vulcanizates filled with cellulose or hydroxyapatite. Phumnok et al. [94] used a ball mill to break up the silica particles and obtain their homogeneous dispersion, resulting in the increased tensile strength of the vulcanizates to about 20 MPa, and with increasing the silica loading, this parameter was improved to 30 MPa. Therefore, it is known that the parameters of the given composites should not be compared in every case. It is generally known that biocomposites with biomass achieve worse useful properties compared to rubber composites filled with commercially used active fillers, i.e., silica or carbon black. However, it is worth mentioning that owing to the production of biocomposites using biomass, sustainable waste management is carried out in accordance with existing pro-ecology trends. Although the biocomposites based on ground eggshells produced by us did not achieve such high mechanical parameters, they are worth producing in order to manage waste and obtain rubber products characterized by physico-chemical properties at a sufficient level to be technologically used.

## 4. Conclusions

Ground eggshells can be successfully applied as inactive biofiller of NR composites, alternatively to commonly used inactive fillers, e.g., chalk or talc. Imidazolium ILs, ammonium surfactant, and silanes can be used for improving the dispersion of ground eggshells in the elastomer matrix. Their application affected the cure characteristics and crosslink density and improved the functional properties of the NR vulcanizates. Thus, it was possible to manufacture not only the environmentally friendly, biodegradable rubber products but also adapting their properties to specific needs. Moreover, using ground eggshells as inactive biofiller of NR composites created opportunities for the management of the bio-waste of animal origin from the food industry. After drying and grinding, eggshells waste can be easily introduced to the rubber compounds.

Vulcanizates filled with ground eggshells with the addition of CTAB and ILs exhibited better tensile properties than the unfilled benchmark. It was a consequence of the higher crosslink density of these vulcanizates and from the homogenous dispersion of ground eggshells in the elastomer matrix, which was mainly due to the acting of additives used. NR vulcanizates filled with eggshells exhibited significantly lower elongation at break than the unfilled benchmark, which resulted from the increased stiffness due to the introduction of biofiller into the elastomer matrix and increased crosslink density of the vulcanizates. On the other hand, ground eggshells and applied additives did not significantly affect the thermal stability of the NR vulcanizates and their ability to dampen vibrations.

Eggshell-filled vulcanizates showed enhanced resistance to thermo-oxidative aging compared to the unfilled benchmark, which is an additional benefit of using this bio-waste as an inactive biofiller for NR composites.

## Figures and Tables

**Table 1:** General recipes of the unfilled NR compounds used in this study; phr, parts per hundred of rubber.

Compound	Unfilled Sample	CTAB	BmiCl	DmiBr
NR	100	100	100	100
ZnO	5	5	5	5
St.A.	1	1	1	1
Sulfur	2	2	2	2
MBT	2	2	2	2
CTAB	-	2	-	-
BmiCl	-	-	2	-
DmiBr	-	-	-	2

**Table 2:** General recipes of the eggshells-filled NR compounds used in this study; phr, parts per hundred of rubber.

Compound	10ES	20ES	30ES	40ES	40ES /APTES	40ES /TESPTS	40ES/ CTAB	40ES/ BmiCl	40ES /DmiBr
NR	100	100	100	100	100	100	100	100	100
ZnO	5	5	5	5	5	5	5	5	5
St.A.	1	1	1	1	-	-	-	-	-
Sulfur	2	2	2	2	2	2	2	2	2
MBT	2	2	2	2	2	2	2	2	2
ES	10	20	30	40	40	40	40	40	40
APTES	-	-	-	-	2	-	-	-	-
TESPTS	-	-	-	-	-	2	-	-	-
CTAB	-	-	-	-	-	-	2	-	-
BmiCl	-	-	-	-	-	-	-	2	-
DmiBr	-	-	-	-	-	-	-	-	2

**Table 3:** Onset temperature of thermal decomposition (T_5%_), DTG peak temperature (T_DTG_), and total mass loss (∆m) during decomposition of pure eggshells and CaCO_3_ (SD: T_5%_ ± 1.3 °C; T_DTG_ ± 1.2 °C; ∆m ± 1.2%).

Biofiller	T_5%_ (°C)	T_DTG_ (°C)	∆m_25–650 °C_ (%)	∆m_650–1100 °C_ (%)	Residue at 1100 °C (%)
Pure eggshells	403	836	6.3	42.3	51.4
CaCO_3_	667	829	3.9	38.9	57.2

**Table 4:** Cure characteristics of NR compounds at 160 °C (S_min_—minimum torque, ΔS—increment of torque during vulcanization, t_02_—scorch time, t_90_—optimal vulcanization time. SD: S_min_ ± 0.2 dNm, ΔS ± 0.5 dNm; t_02_ ± 0.2 min; t_90_ ± 0.5 min; ν_t_ ± 0.2 × 10^−5^ mol/cm^3^).

NR Composite	S_min_ (dNm)	∆S (dNm)	t_02_ (min)	t_90_ (min)	ν_t_ × 10^−5^ (mol/cm^3^)
Unfilled sample	0.4	5.1	1	2	0.9
CTAB	0.4	7.4	1	2	1.6
BmiCl	0.4	6.9	1	2	1.4
DmiBr	0.4	7.1	1	2	1.4
10ES	0.4	5.9	1	2	1.0
20ES	0.6	6.8	1	2	1.1
30ES	0.6	7.2	1	2	1.2
40ES	0.4	7.4	1	2	1.2
40ES/APTES	0.6	7.5	1	2	1.2
40ES/TESPTS	0.3	6.7	1	3	1.1
40ES/CTAB	0.3	9.2	1	3	1.5
40ES/BmiCl	0.3	8.6	1	2	1.3
4ES/DmiBr	0.2	9.5	1	2	1.4

**Table 5:** Vulcanization temperature (T_vul_) and enthalpy (ΔH_vul_) of NR composites examined by DSC (SD: T_vul_ ± 2 °C; ΔH_vul_ ± 1.2 J/g).

NR Composite	T_vul_ (°C)	−∆H_vul_ (J/g)
Unfilled sample	174–210	13.8
CTAB	145–218	10.9
BmiCl	138–218	10.1
DmiBr	138–220	9.9
10ES	136–213	9.2
20ES	137–229	11.6
30ES	138–208	10.6
40ES	138–208	7.6
40ES/APTES	153–204	11.6
40ES/TESPTS	158–216	7.6
40ES/CTAB	140–214	7.0
40ES/BmiCl	135–221	7.0
40ES/DmiBr	140–222	7.3

**Table 6:** Tensile properties and hardness of NR vulcanizates (SE_300_—stress at a relative elongation of 300%; TS—tensile strength; E_b_—elongation at break; H—hardness).

NR Vulcanizate	SE_300_ (MPa)	TS (MPa)	E_b_ (%)	H (Shore A)
Unfilled sample	1.2 ± 0.1	9.6 ± 0.3	820 ± 20	31 ± 1
10ES	1.4 ± 0.1	8.6 ± 0.8	669 ± 20	34 ± 1
20ES	1.7 ± 0.1	10.4 ± 0.3	634 ± 25	37 ± 1
30ES	1.7 ± 0.2	11.1 ± 0.8	613 ± 23	38 ± 1
40ES	1.5 ± 0.1	9.3 ± 0.7	674 ± 21	38 ± 1
40ES/APTES	1.8 ± 0.1	7.7 ± 0.6	602 ± 16	37 ± 1
40ES/TESPTS	1.3 ± 0.1	8.0 ± 0.6	696 ± 16	37 ± 1
40ES/CTAB	2.4 ± 0.1	11.9 ± 0.8	569 ± 26	44 ± 1
40ES/BmiCl	2.0 ± 0.1	11.4 ± 0.7	627 ± 23	42 ± 1
40ES/DmiBr	2.2 ± 0.1	12.0 ± 0.4	619 ± 17	43 ± 1

**Table 7:** Glass transition temperature (T_g_) and loss factor (tan δ) of NR vulcanizates measured by DMA.

NR Vulcanizate	T_g_ (°C)	tan δ_Tg_ (−)	tan δ_25 °C_ (−)	tan δ_60 °C_ (−)
Unfilled sample	−68 ± 1	2.7± 0.1	0.06 ± 0.02	0.05 ± 0.01
CTAB	−69 ± 1	2.3 ± 0.1	0.05 ± 0.02	0.04 ± 0.01
BmiCl	−69 ± 1	2.6 ± 0.1	0.05 ± 0.02	0.04 ± 0.01
DmiBr	−69 ± 1	2.6 ± 0.1	0.05 ± 0.02	0.04 ± 0.01
10ES	−69 ± 1	2.6 ± 0.1	0.05 ± 0.02	0.03 ± 0.01
20ES	−69 ± 1	2.6 ± 0.1	0.05 ± 0.02	0.03 ± 0.01
30ES	−67 ± 1	2.4 ± 0.1	0.05 ± 0.02	0.03 ± 0.01
40ES	−69 ± 1	2.4 ± 0.1	0.05 ± 0.02	0.04 ± 0.01
40ES/APTES	−69 ± 1	2.5 ± 0.1	0.06 ± 0.02	0.04 ± 0.01
40ES/TESPTS	−69 ± 1	2.6 ± 0.1	0.06 ± 0.02	0.03 ± 0.01
40ES/CTAB	−69 ± 1	2.3 ± 0.1	0.04 ± 0.02	0.03 ± 0.01
40ES/BmiCl	−70 ± 1	2.6 ± 0.1	0.03 ± 0.02	0.03 ± 0.01
40ES/DmiBr	−69 ± 1	2.6 ± 0.1	0.03 ± 0.02	0.03 ± 0.01

**Table 8:** Thermo-oxidative aging factor (A_f_) of NR vulcanizates.

NR Composite	A_f_ (−)
Unfilled sample	0.5 ± 0.1
10ES	0.7 ± 0.1
20ES	0.7 ± 0.1
30ES	0.7 ± 0.1
40ES	0.7 ± 0.1
40ES/APTES	0.7 ± 0.1
40ES/TESPTS	0.7 ± 0.1
40ES/CTAB	0.9 ± 0.1
40ES/BmiCl	0.8 ± 0.1
40ES/DmiBr	0.8 ± 0.1

**Table 9:** Onset temperature of thermal decomposition (T_5%_), DTG peak temperature (T_DTG_), and total mass loss (∆m) during decomposition of NR composites filled with eggshells (SD: T_5%_ ± 1.2 °C; T_DTG_ ± 1.3 °C; ∆m ± 1.2%).

NR Vulcanizate	T_5%_ (°C)	T_DTG_ (°C)	∆m_25–600 °C_ (%)	∆m_600–900 °C_ (%)	Residue at 900 °C %)
Unfilled sample	322	398	97.1	0.9	1.9
10ES	319	396	87.2	3.0	9.8
20ES	320	395	81.2	4.6	14.2
30ES	324	395	77.6	5.8	16.6
40ES	328	396	71.3	9.7	19.0
40ES/APTES	331	395	70.9	10.0	19.1
40ES/TESPTS	322	395	72.3	10.6	17.1
40ES/CTAB	325	395	71.1	10.3	18.6
40ES/BmiCl	325	397	70.7	10.0	19.3
40ES/DmiBr	324	396	69.7	10.9	19.4

## Data Availability

The data presented in this study are available on request from the corresponding author.

## Supplementary Materials

The following supporting information can be downloaded at: https://www.mdpi.com/article/10.3390/ma16082988/s1, One-way ANOVA analysis of the effect of ground eggshell content and different additives (silanes, CTAB, ILs) on the cure characteristics and properties of natural rubber biocomposites Tables S1: ANOVA for minimum torque (Smin) of NR biocomposites. Table S2: ANOVA for torque increase (ΔS) of NR biocomposites. Table S3: ANOVA for optimal vulcanization time (t_90_) of NR biocomposites. Table S4: ANOVA for crosslink density (ν_t_) of NR biocomposites. Table S5: ANOVA for onset vulcanization temperature of NR biocomposites. Table S6: ANOVA for vulcanization enthalpy (ΔH) of NR biocomposites. Table S7: ANOVA for stress at a relative elongation of 300% (SE_300_) of NR biocomposites. Table S8: ANOVA for tensile strength (TS) of NR biocomposites. Table S9: ANOVA for elongation at break (EB) of NR biocomposites. Table S10: ANOVA for hardness of NR biocomposites. Table S11: ANOVA for glass transition temperature (T_g_) of NR biocomposites. Table S12: ANOVA for loss factor at glass transition temperature (tanδ_Tg_) of NR biocomposites. Table S13: ANOVA for loss factor at 25°C (tanδ_25°C_) of NR biocomposites. Table S14: ANOVA for loss factor at 60°C (tanδ_60°C_) of NR biocomposites. Table S15: ANOVA for aging factor (A_f_) of NR biocomposites. Table S16: ANOVA for onset decomposition temperature (T_5%_) of NR biocomposites. Table S17: ANOVA for DTG peak temperature (T_DTG_) of NR biocomposites. Table S18: ANOVA for mass loss in the temperature range of 25-600°C (Δm_25-600°C_) of NR biocomposites. Table S19: ANOVA for mass loss in the temperature range of 600-900°C (Δm_600-900°C_) of NR biocomposites. Table S20: ANOVA for decomposition residue at 900 °C of NR biocomposites.

## Abbreviations

*A_f_*, aging factor; APTES, (3-aminopropyl)-triethoxysilane; BmiCl, 1-butyl-3-methylimidazolium chloride; CaCO_3_, calcium carbonate; CaO, calcium oxide; CTAB, cetyltrimethylammonium bromide; DMA, dynamic mechanical analysis; DmiBr, 1-decyl-3-methylimidazolium bromide; DSC, differential scanning calorimetry; DTG, derivative thermogravimetry; *E_b_*, elongation at break; ES, eggshells; FTIR, Fourier transform infrared spectroscopy; H, hardness; ΔH_vul_, enthalpy of vulcanization; ILs, ionic liquids; ∆m, total mass loss; MBT, 2-mercaptobenzothiazole; NR, natural rubber; ∆S, torque increase; S_min_, minimum torque; SE_300_, stress at a relative elongation of 300%; SEM, scanning electron microscopy; St. A., stearic acid; t_02_, scorch time; T_05_, onset temperature of thermal decomposition; t_90_, optimal vulcanization time; T_DTG_, derivative thermogravimetric peak temperature; tan δ, loss factor; TESPTS, bis[3-(triethoxysilyl)propyl] tetrasulfide; TG, thermogravimetry; T_g_, glass transition temperature; *TS*, tensile strength; T_vul_, vulcanization temperature; ν_t_, crosslink density; *χ*, Huggins parameter; ZnO, zinc oxide.
