# Supplementary material for: Potential Utilization of Ground Eggshells as a Biofiller for Natural Rubber Biocomposites
Source: Materials (Basel). 2023 Apr 9;16(8):2988. doi: 10.3390/ma16082988 (PMC10143961; doi:10.3390/ma16082988)
Supplement: Supplementary file 1 [file materials-16-02988-s001.zip › materials-2284184-supplementary.pdf]

Supplementary Materials

Potential Utilization of Ground Eggshells as a Biofiller for Natural Rubber Biocomposites

Supplementary Material – one-way ANOVA analysis of the effect of ground eggshells content and different additives (silanes, CTAB, ILs) on the cure characteristics and properties of natural rubber biocomposites.

The significance level was taken as P\0.05 (5%), i.e., for a level of confidence of 95%.

Table S1. ANOVA for minimum torque (S_{min}) of NR biocomposites

SUMMATION

Groups	*Counter*	*Sum*	*Avarage*	*Variance*
No additives	5	2.4	0.48	0.012
With additives	5	1.7	0.34	0.023

VARIANCE ANALYSIS

Source of variance	*SS*	*df*	*MS*	F α = 5%	P /%	*Test F*
Between groups	0.049	1	0.049	2.80*	0.13	5.32
Within groups	0.140	8	0.018			
Total	0.189	9				

df - degrees of freedom, SS - sum of squares, MS – mean square, P - percentage of contribution
* The means difference is not significant at the 0.05 level.

Table S2. ANOVA for torque increase (ΔS) of NR biocomposites

SUMMATION

Groups	*Counter*	*Sum*	*Avarage*	*Variance*
No additives	5	32.3	6.46	0.958
With additives	5	41.5	8.30	1.385

VARIANCE ANALYSIS

Source of variance	*SS*	*df*	*MS*	F α = 5%	P /%	*Test F*
Between groups	8.464	1	8.464	7.22*	0.03	5.32
Within groups	9.372	8	1.172			
Total	17.836	9				

df - degrees of freedom, SS - sum of squares, MS – mean square, P - percentage of contribution
* The means difference is significant at the 0.05 level.

Table S3. ANOVA for optimal vulcanization time (t_{90}) of NR biocomposites

SUMMATION

Groups	*Counter*	*Sum*	*Avarage*	*Variance*
No additives	5	10	2.0	0.0
With additives	5	12	2.4	0.3

VARIANCE ANALYSIS

Source of variance	*SS*	*df*	*MS*	F α = 5%	P /%	*Test F*
Between groups	0.400	1	0.400	2.67*	0.14	5.32
Within groups	1.200	8	0.150			
Total	1.600	9				

df - degrees of freedom, SS - sum of squares, MS – mean square, P - percentage of contribution
* The means difference is not significant at the 0.05 level.

Table S4. ANOVA for crosslink density (ν_t) of NR biocomposites

SUMMATION

Groups	*Counter*	*Sum*	*Avarage*	*Variance*
No additives	5	5.4	1.08	0.017
With additives	5	6.5	1.30	0.025

VARIANCE ANALYSIS

Source of variance	*SS*	*df*	*MS*	F α = 5%	P /%	*Test F*
Between groups	0.121	1	0.121	5.76*	0.04	5.32
Within groups	0.168	8	0.021			
Total	1.600	9				

df - degrees of freedom, SS - sum of squares, MS – mean square, P - percentage of contribution
* The means difference is significant at the 0.05 level.

Table S5. ANOVA for onset vulcanization temperature of NR biocomposites

SUMMATION

Groups	*Counter*	*Sum*	*Avarage*	*Variance*
No additives	5	723	144.6	270.8
With additives	5	726	145.2	95.7

VARIANCE ANALYSIS

Source of variance	*SS*	*df*	*MS*	F α = 5%	P /%	*Test F*
Between groups	0.9	1	0.90	0.004*	0.95	5.32
Within groups	1466	8	183.25			
Total	1466.9	9				

df - degrees of freedom, SS - sum of squares, MS – mean square, P - percentage of contribution
* The means difference is not significant at the 0.05 level.

Table S6. ANOVA for vulcanization enthalpy (ΔH) of NR biocomposites

SUMMATION

Groups	*Counter*	*Sum*	*Avarage*	*Variance*
No additives	5	52.8	10.56	5.548
With additives	5	40.5	8.10	3.890

VARIANCE ANALYSIS

Source of variance	SS	df	MS	F α = 5%	P /%	Test F
Between groups	15.129	1	15.129	3.21*	0.11	5.32
Within groups	37.752	8	4.719			
Total	52.881	9				

df - degrees of freedom, SS - sum of squares, MS – mean square, P - percentage of contribution
* The means difference is not significant at the 0.05 level.

Table S7. ANOVA for stress at a relative elongation of 300% (SE_{300}) of NR biocomposites

SUMMATION

Groups	Counter	Sum	Avarage	Variance
No additives	5	7.5	1.50	0.045
With additives	5	9.7	1.94	0.178

VARIANCE ANALYSIS

Source of variance	SS	df	MS	F α = 5%	P /%	Test F
Between groups	0.484	1	0.484	4.34*	0.07	5.32
Within groups	0.892	8	0.112			
Total	1.376	9				

df - degrees of freedom, SS - sum of squares, MS – mean square, P - percentage of contribution
* The means difference is not significant at the 0.05 level.

Table S8. ANOVA for tensile strength (TS) of NR biocomposites

SUMMATION

Groups	Counter	Sum	Avarage	Variance
No additives	5	49	9.8	0.945
With additives	5	51	10.2	4.665

VARIANCE ANALYSIS

Source of variance	SS	df	MS	F α = 5%	P /%	Test F
Between groups	0.40	1	0.400	0.14*	0.72	5.32
Within groups	22.44	8	2.805			
Total	22.84	9				

df - degrees of freedom, SS - sum of squares, MS – mean square, P - percentage of contribution
* The means difference is not significant at the 0.05 level.

Table S9. ANOVA for elongation at break (EB) of NR biocomposites

SUMMATION

Groups	Counter	Sum	Avarage	Variance
No additives	5	3410	682.0	6585.5
With additives	5	3113	622.6	2179.3

VARIANCE ANALYSIS

Source of variance	*SS*	*df*	*MS*	F α = 5%	P /%	*Test F*
Between groups	8820.9	1	8820.9	2.01*	0.19	5.32
Within groups	35059.2	8	4382.4			
Total	43880.1	9				

df - degrees of freedom, SS - sum of squares, MS – mean square, P - percentage of contribution
* The means difference is not significant at the 0.05 level.

Table S10. ANOVA for hardness of NR biocomposites

SUMMATION

Groups	*Counter*	*Sum*	*Avarage*	*Variance*
No additives	5	178	35.6	9.3
With additives	5	203	40.6	11.3

VARIANCE ANALYSIS

Source of variance	*SS*	*df*	*MS*	F α = 5%	P /%	*Test F*
Between groups	62.5	1	62.5	6.07*	0.04	5.32
Within groups	82.4	8	10.3			
Total	144.9	9				

df - degrees of freedom, SS - sum of squares, MS – mean square, P - percentage of contribution
* The means difference is significant at the 0.05 level.

Table S11. ANOVA for glass transition temperature (T_g) of NR biocomposites

SUMMATION

Groups	*Counter*	*Sum*	*Avarage*	*Variance*
No additives	5	342	68.4	0.8
With additives	5	346	69.2	0.2

VARIANCE ANALYSIS

Source of variance	*SS*	*df*	*MS*	F α = 5%	P /%	*Test F*
Between groups	1.6	1	1.6	3.20*	0.11	5.32
Within groups	4.0	8	0.5			
Total	5.6	9				

df - degrees of freedom, SS - sum of squares, MS – mean square, P - percentage of contribution
* The means difference is not significant at the 0.05 level.

Table S12. ANOVA for loss factor at glass transition temperature ($\tan\delta_{Tg}$) of NR biocomposites

SUMMATION

Groups	*Counter*	*Sum*	*Avarage*	*Variance*
No additives	5	12.7	2.54	0.018
With additives	5	12.6	2.52	0.017

VARIANCE ANALYSIS

Source of variance	*SS*	*df*	*MS*	F α = 5%	P /%	*Test F*
Between groups	0.001	1	0.001	0.06*	0.82	5.32
Within groups	0.140	8	0.018			
Total	0.141	9				

df - degrees of freedom, SS - sum of squares, MS – mean square, P - percentage of contribution
* The means difference is not significant at the 0.05 level.

Table S13. ANOVA for loss factor at 25°C ($tan\delta_{25°C}$) of NR biocomposites

SUMMATION

Groups	*Counter*	*Sum*	*Avarage*	*Variance*
No additives	5	0.26	0.052	0.00002
With additives	5	0.22	0.044	0.00023

VARIANCE ANALYSIS

Source of variance	*SS*	*df*	*MS*	F α = 5%	P /%	*Test F*
Between groups	0.00016	1	0.00016	1.28*	0.29	5.32
Within groups	0.00100	8	0.00013			
Total	0.00116	9				

df - degrees of freedom, SS - sum of squares, MS – mean square, P - percentage of contribution
* The means difference is not significant at the 0.05 level.

Table S14. ANOVA for loss factor at 60°C ($tan\delta_{60°C}$) of NR biocomposites

SUMMATION

Groups	*Counter*	*Sum*	*Avarage*	*Variance*
No additives	5	0.18	0.036	0.00008
With additives	5	0.16	0.032	0.00002

VARIANCE ANALYSIS

Source of variance	*SS*	*df*	*MS*	F α = 5%	P /%	*Test F*
Between groups	0.00004	1	0.00004	0.80*	0.40	5.32
Within groups	0.00040	8	0.00005			
Total	0.00044	9				

df - degrees of freedom, SS - sum of squares, MS – mean square, P - percentage of contribution
* The means difference is not significant at the 0.05 level.

Table S15. ANOVA for aging factor (A_f) of NR biocomposites

SUMMATION

Groups	*Counter*	*Sum*	*Avarage*	*Variance*
No additives	5	3.3	0.66	0.008
With additives	5	3.9	0.78	0.007

VARIANCE ANALYSIS

Source of variance	*SS*	*df*	*MS*	F α = 5%	P /%	*Test F*
Between groups	0.036	1	0.036	4.80*	0.06	5.32
Within groups	0.060	8	0.008			
Total	0.096	9				

df - degrees of freedom, SS - sum of squares, MS – mean square, P - percentage of contribution
* The means difference is not significant at the 0.05 level.

Table S16. ANOVA for onset decomposition temperature ($T_{5\%}$) of NR biocomposites

SUMMATION

Groups	*Counter*	*Sum*	*Avarage*	*Variance*
No additives	5	1613	322.6	12.8
With additives	5	1627	325.4	11.3

VARIANCE ANALYSIS

Source of variance	*SS*	*df*	*MS*	F α = 5%	P /%	*Test F*
Between groups	19.6	1	19.60	1.63*	0.24	5.32
Within groups	96.4	8	12.05			
Total	116.0	9				

df - degrees of freedom, SS - sum of squares, MS – mean square, P - percentage of contribution
* The means difference is not significant at the 0.05 level.

Table S17. ANOVA for DTG peak temperature (T_{DTG}) of NR biocomposites

SUMMATION

Groups	*Counter*	*Sum*	*Avarage*	*Variance*
No additives	5	1980	396.0	1.5
With additives	5	1978	395.6	0.8

VARIANCE ANALYSIS

Source of variance	*SS*	*df*	*MS*	F α = 5%	P /%	*Test F*
Between groups	0.4	1	0.40	0.35*	0.57	5.32
Within groups	9.2	8	1.15			
Total	9.6	9				

df - degrees of freedom, SS - sum of squares, MS – mean square, P - percentage of contribution
* The means difference is not significant at the 0.05 level.

Table S18. ANOVA for mass loss in the temperature range of 25-600°C ($\Delta m_{25\text{-}600°C}$) of NR biocomposites

SUMMATION

Groups	*Counter*	*Sum*	*Avarage*	*Variance*
No additives	5	414.4	82.88	96.417
With additives	5	354.7	70.94	0.868

VARIANCE ANALYSIS

Source of variance	*SS*	*df*	*MS*	F α = 5%	P /%	*Test F*
Between groups	356.41	1	356.41	7.33*	0.03	5.32
Within groups	389.14	8	48.64			
Total	745.55	9				

df - degrees of freedom, SS - sum of squares, MS – mean square, P - percentage of contribution
* The means difference is significant at the 0.05 level.

Table S19. ANOVA for mass loss in the temperature range of 600-900°C ($\Delta m_{600\text{-}900°C}$) of NR biocomposites

SUMMATION

Groups	*Counter*	*Sum*	*Avarage*	*Variance*
No additives	5	24.0	4.80	10.875
With additives	5	51.8	10.36	0.153

VARIANCE ANALYSIS

Source of variance	*SS*	*df*	*MS*	F α = 5%	P /%	*Test F*
Between groups	77.28	1	77.28	14.01*	0.006	5.32
Within groups	44.11	8	5.51			
Total	121.39	9				

df - degrees of freedom, SS - sum of squares, MS – mean square, P - percentage of contribution
* The means difference is significant at the 0.05 level.

Table S20. ANOVA for decomposition residue at 900°C of NR biocomposites

SUMMATION

Groups	*Counter*	*Sum*	*Avarage*	*Variance*
No additives	5	61.5	12.3	45.350
With additives	5	93.5	18.7	0.895

VARIANCE ANALYSIS

Source of variance	*SS*	*df*	*MS*	F α = 5%	P /%	*Test F*
Between groups	102.40	1	102.40	4.43*	0.07	5.32
Within groups	184.98	8	23.12			
Total	287.38	9				

df - degrees of freedom, SS - sum of squares, MS – mean square, P - percentage of contribution
* The means difference is not significant at the 0.05 level.
